# *n*-3 PUFA Promotes Ferroptosis in PCOS GCs by Inhibiting YAP1 through Activation of the Hippo Pathway

**DOI:** 10.3390/nu15081927

**Published:** 2023-04-16

**Authors:** Peiwen Zhang, Yuheng Pan, Shuang Wu, Yuxu He, Jinyong Wang, Lei Chen, Shunhua Zhang, Hui Zhang, Ye Zhao, Lili Niu, Mailin Gan, Yan Wang, Linyuan Shen, Li Zhu

**Affiliations:** 1 Key Laboratory of Livestock and Poultry Multi-Omics, Ministry of Agriculture and Rural Affairs, College of Animal and Technology, Sichuan Agricultural University, Chengdu 611130, China; zpw1995@stu.sicau.edu.cn (P.Z.); panyuheng@stu.sicau.edu.cn (Y.P.); wushuang@stu.sicau.edu.cn (S.W.); heyuxu@stu.sicau.edu.cn (Y.H.); chenlei815918@sicau.edu.cn (L.C.); 14081@sicau.edu.cn (S.Z.); zhye@sicau.edu.cn (Y.Z.); niulili@sicau.edu.cn (L.N.); ganmailin@sicau.edu.cn (M.G.); zhuli@sicau.edu.cn (L.Z.); 2 Farm Animal Genetic Resource Exploration and Innovation Key Laboratory of Sichuan Province, Sichuan Agricultural University, Chengdu 611130, China; 3 Chongqing Academy of Animal Science, Chongqing 402460, China; kingyongwang7704@163.com; 4 Sichaun Center for Animal Disease Control, Chengdu 610041, China; zhanghui7903@163.com

**Keywords:** PCOS, ferroptosis, YAP1, *n*-3 PUFA

## Abstract

Polycystic ovary syndrome (PCOS) is an endocrine disorder characterized by hyperandrogenemia with multiple suspended sinus follicles, thickened cortical tissue, and excessive proliferation of ovarian granulosa cells that severely affects the fertility and quality of life of women. The addition of *n*-3 PUFA to the diet may slightly reduce body weight and greatly alleviate disturbed blood hormone levels in PCOS mice. We treated KGN as a cell model for *n*-3 PUFA addition and showed that *n*-3 PUFA inhibited the proliferation of GCs and promoted ferroptosis in ovarian granulosa cells. We used CCK-8, fluorescence quantitative transmission electron microscopy experiments and ferroptosis marker gene detection and other methods. Furthermore, *n*-3 PUFA was found to promote YAP1 exocytosis by activating Hippo and weakening the cross-talk between YAP1 and Nrf2 by activating the Hippo signaling pathway. In this study, we found that *n*-3 PUFA inhibited the over proliferation of granulosa cells in ovarian follicles by activating Hippo, promoting YAP1 exocytosis, weakening the cross-talk between YAP1 and Nrf2, and ultimately activating the ferroptosis sensitivity of ovarian granulosa cells. We demonstrate that *n*-3 PUFA can alleviate the hormonal and estrous cycle disorder with PCOS by inhibiting the YAP1-Nrf2 crosstalk that suppresses over proliferating ovarian granulosa cells and promotes iron death in GCs. These findings reveal the molecular mechanisms by which *n*-3 PUFA attenuates PCOS and identify YAP1-Nrf2 as a potential therapeutic target for regulation granulosa cells in PCOS.

## 1. Introduction

Polycystic ovary syndrome, a condition affecting women of reproductive age, is an endocrine disorder notorious for hindering the chances of normal conception [1,2]. The clinical manifestation of polycystic ovaries is characterized by a highly heterogeneous presentation that includes insulin resistance, hyperandrogenemia, polycystic ovarian lesions, sporadic ovulation or persistent anovulation, and endocrine disorders. Notably, the prevalence of PCOS in women of childbearing age ranges between 6% and 10%, but the prevalence is significantly higher in women with underlying conditions than in the general population [3]. The clinical sequelae of PCOS are severe and are associated with insulin resistance (type 2 diabetes), depression, anxiety, and other debilitating symptoms that have a profound negative impact on the quality of life and psychological well-being of the patient [4]. While current treatments for PCOS mainly include pharmacological interventions aimed at re-establishing the menstrual cycle, providing emotional relief, and improving lifestyle, these treatments only target the clinical symptoms of PCOS and, unfortunately, yield unsatisfactory results [5]. Therefore, understanding the complex physiological mechanisms of PCOS pathogenesis and treatment has become a hot topic of research in gynecological endocrine diseases. In short, given the perplexing nature of the disorder’s presentation and its impact on reproductive health, there is an urgent need to advance the current understanding of PCOS to improve the health and well-being of affected women.

The etiology of polycystic ovary syndrome (PCOS) is intrinsically linked to the patient’s genetic single nucleotide polymorphisms (SNPs) and their ambient environment, rendering its pathogenesis rather complex [6]. PCOS patients, however, may alleviate their clinical symptoms and curb the disease progression through prudent modifications to their daily dietary and lifestyle routines [7]. It has been reported that the consumption of certain small molecules of food origin, e.g., curcumin and vitamin E, can ameliorate PCOS symptoms. Moreover, consumption of *n*-3 polyunsaturated fatty acids (PUFA) can provide a therapeutic avenue, owing to their multifarious anti-inflammatory, anti-hyperlipidemic, and insulin resistance properties [8]. Ensuring a daily intake of a certain threshold of polyunsaturated fatty acids in PCOS patients has been shown to significantly improve the metabolic syndrome associated with PCOS [9]. Furthermore, *n*-3 PUFA may participate in the regulation of the arachidonic acid derivative process in the organism’s lipid metabolism, ultimately downregulating the expression of inflammatory factors in vivo [10]. Remarkably, it has been shown that *n*-3 PUFA intake can markedly suppress the arachidonic acid COX-derived PG eicosanoid group levels in obese individuals, which otherwise propagate the activation of a chronic inflammatory environment [11,12].

Ferroptosis, a fresh variant of necrotic cellular demise, differs significantly from apoptotic focal autophagy. The intricacies of ferroptosis have been widely scrutinized in various studies, demonstrating its involvement in the pathogenesis of diverse diseases, including but not limited to neurodegenerative disorders, diabetes, hypertension, stroke, and cancer [13]. The utilization of treatments that promote ferroptosis susceptibility in disease models has been observed to curb the progression of specific maladies, indicating that ferroptosis could be a key factor in developing therapeutic and targeted drugs for various diseases [14,15,16].To cite an instance, Emeline Dierge et al. [17]. showcased how the selective induction of ferroptosis in cancer cells under acidotic conditions in tumor cells could halt tumor growth in the treated group of mice enriched with *n*-3 PUFA in the diet. Additionally, Chen et al. [18]. found that the exogenous addition of *n*-3 PUFA could significantly hinder tumor growth by enhancing the degradation of glutathione in multiple myeloma cells, thus promoting ferroptosis and augmenting sensitivity to drug treatment.

Iron, an essential trace element in mammals, plays a crucial role in various biological processes such as electron transfer, cell proliferation, and protein synthesis [19]. However, when the organism’s iron metabolism is disturbed, an influx of free ferrous ions causes ROS production catalyzed by the Fenton reaction. The surplus of ROS can lead to severe damage to cell membranes, organelles, and nuclei, and excessive ROS levels can further drive lipid peroxidation, ultimately leading to cellular ferroptosis.

Interestingly, a strong connection exists between *n*-3 PUFA and iron metabolism. Excessive iron intake could result in a phenotype of enhanced oxidative stress in the liver [19]. It has been shown that iron metabolism is disturbed in serum samples from patients with NAFLD, and *n*-3 PUFA levels are depleted in the liver [20]. Furthermore, excessive iron intake leads to a decrease in the activity of delta-5 and delta-6 [20] desaturases, which synthesize *n*-3 PUFA as well as n-6 PUFA. Consequently, a direct or indirect regulatory relationship between iron and *n*-3 PUFA is not difficult to perceive [11]. Nonetheless, the precise molecular mechanisms of regulation responsible for the effects of *n*-3 PUFA intake on the clinical symptoms of polycystic ovary syndrome remain unreported.

The Hippo signaling pathway, a highly conserved pathway across various species, plays a crucial role in the regulation of proliferation and differentiation of multiple cell types [21]. As a result, a large body of research now suggests that this pathway is involved in embryonic development, cancer development, as well as the regulation of organ size and tissue overgrowth [22]. When it comes to follicle development, the Hippo pathway is of particular relevance, as activation of the pathway leads to the alteration of nuclear positioning of YAP1 to perform different regulatory functions.

Interestingly, the expression of both YAP1 and phosphorylated-YAP1 has been detected in the mammalian ovary [23]. However, YAP1 expression is mainly distributed in the nucleus of ovarian granulosa cells during the primary to preovulatory stage. In contrast, in differentiated luteal cells, YAP1 is phosphorylated and retained in the cytoplasm, indicating that YAP1 expression and localization is highly dynamic and regulated.

Moreover, activation of YAP1 has been shown to promote the expression of NRF2, highlighting the intricate crosstalk between YAP1 and NRF2 [24]. Overall, the Hippo signaling pathway and its relationship to YAP1 and NRF2 are essential areas of research with significant implications for various fields, including embryonic development, cancer, and organ size regulation.

The domain of PCOS has been a perplexing and enigmatic subject in the medical world. Despite numerous investigations, the association between PCOS and ferroptosis has been vastly underreported. It is noteworthy that the modulatory mechanism of ovarian granulosa cells in PCOS by *n*-3 PUFA remains a profound mystery [25]. Thus, we embarked on an expedition to unravel the intricacies of the molecular mechanisms underlying the favorable effects of *n*-3 PUFA on the treatment of PCOS. In this expedition, we employed a multifaceted approach that involved investigating the signaling pathways targeted for regulation. Through our research, we unearthed the astounding fact that *n*-3 PUFA has an inhibitory effect on the proliferation of ovarian granulosa cells in PCOS, which is a direct consequence of promoting ferroptosis of ovarian granulosa cells. This effect culminates in the alleviation of the debilitating clinical symptoms associated with PCOS.

## 2. Materials and Methods

### 2.1. Ethical Approval of the Study

Mice were handled and experimental procedures were in accordance with the regulations of the Animal Ethics and Welfare Committee of Sichuan Agricultural University and approved by the College of Animal Science and Technology of Sichuan Agricultural University.

### 2.2. Care of Experimental Animals

The experimental mice were housed in an IVC mouse housing system with a temperature of 23 °C and a 12-h light-dark cycle. Mice were allowed to eat and drink freely. The experimental protocol was approved by the Animal Ethics and Welfare Committee of Sichuan Agricultural University, review number 2019102010.

### 2.3. PCOS Mice Modelling and *n*-3 PUFA

Three-week-old ICR mice were injected with DHEA (6 mg/100 g body weight) for 21 consecutive days. We weighed an appropriate amount of DHEA and dissolved it in injectable grade soybean oil and injected it subcutaneously into the back of mice. DHEA was purchased from Coolaber Company, CD4222, purity >99%, to determine whether the estrous cycle was disrupted by toluidine blue staining of vaginal epithelial cells. Dietary intervention with *n*-3 PUFAs started 21 days after DHEA treatment in mice. Mice were garaged with *n*-3 fatty acids at 0.2 mL each for 60 days (DHA 180 mg/mL EPA 120 mg/mL).

### 2.4. Cell Culture

The KGN cell line was purchased from Pronoxa Biologicals (STR verified) and the medium was purchased from Pronoxa CM-0603.We added 10% fetal bovine serum and a 1% mixture of penicillin and streptomycin to the medium of the KGN cells. All cells were cultured at 37 °C in a 5% CO_2_ cell culture chamber. Cells were digested with trypsin and spread in cell culture plates at a density of 100,000 cells per ml, and the experimental treatment was started when the cell density grew to 50–60%. The KGN cell line was supplemented with *n*-3 PUFA at a concentration of 1.8 ng/mL DHA and 1.2 ng/mL EPA for 48 h. We added Fer-1 (Shanghai yuanye Bio-Technology Company, Shanghai, China, S81461, >98%) to the KGNcells, diluted it to a concentration of 20 mM with DMSO, added 1 μL of Fer-1 to 1 mL of medium, and ensured that the final concentration was 20 μM

### 2.5. Observation of the Motility Cycle in Mice

Vaginal epithelial cell sections of mice were prepared daily with PBS to observe the oestrus cycle for 3 oestrus cycles. Then, 10 μL of PBS was gently injected into the vagina of the mice, and the liquid was repeatedly aspirated and applied to slides to dry. The sections were fixed in 95% ethanol, stained with toluidine blue staining solution, and observed under a light microscope.

### 2.6. Detection of Serum Biochemical Indicators

The serum is operated according to the steps of mlbio ELISA kit, and the absorbance value is finally converted to the corresponding concentration of the standard curve (Testosterone ml001948; Estradiol ml001962; LH ml063366; Progesterone ml057778; FSH ml001910; AMH ml037597; Prolactin ml001906).

### 2.7. Immunofluorescence

Add cell fixation solution (the effective ingredient is 4% paraformaldehyde, use 0.1 M phosphate buffer as solvent, pH 7.0–7.5, store at 25 °C) to the cell culture plate containing cell slides, fix at room temperature for 20 min, and replace with Wash 1–2 times with 1× PBS. Add 10% donkey serum or 3% BSA to the cell plate and incubate for 30 min, add YAP1 antibody (1:500 dilution) and phalloidin antibody diluted in the primary antibody diluent, and then incubate overnight at 4 °C. After adding the secondary antibody and incubating for 50 min, DAPI staining was performed for 15 min, and finally the cell slides were observed and photographed under a fluorescent inverted microscope.

### 2.8. Western Blot

We added 100 μL RIPA lysate (containing PMSF and phosphatase inhibitors) to 10^6^ cells, detected the protein concentration with BCA kit (Beyond Biotech Co., Ltd., Kaohsiung City, Taiwan), and denatured the protein with 5× SDS Buffer. Protein denaturation was carried out at 95 °C for 10 min. After treatment, the protein was subjected to SDS PAGE electrophoresis until the bromophenol blue band ran to the bottom of the gel and terminated. The methanol-activated PVDF membrane was covered on the gel for transmembrane experiment, 200 mA for 2 h, and then incubated with primary antibody and secondary antibody. Finally, use ECL to display the color agent to expose the strips and use imageJ to carry out the statistics of the gray scale of the strips.

### 2.9. RNA Extraction and Reverse Transcription

RNA extraction was carried out according to RNAiso (Takara, Tokyo, Japan), and cDNA was made using a PrimeScript TM RT Reagent Kit with gDNA Eraser (TaKaRa, Dalian, China).

### 2.10. Real-Time Fluorescent Quantitative PCR

Fluorescent PCR experiments were performed as in our previous study [26]. The primers used in this study are listed in Table S1.

### 2.11. Transmission Electron Microscopy

The treated cells were collected into cell suspensions (Collect at least 2 × 10^6^ cells), centrifuged at 1500× *g* for 4 min, and the cell pellets were fixed in electron microscope fixative for 4 h and stored at 4 °C. The cells were pre-embedded in agar and fixed in 1% osmium acid prepared in 0.1 M phosphate buffer PB (pH 7.4) for 2 h at room temperature, then dehydrated in 30%-50%-70%-80%-95%-100%-100% alcohol for 20 min each time and 100% acetone twice for 15 min each time.

After the infiltration embedding as well as the polymerisation step, ultra-thin sections were made. The copper mesh was stained in 2% uranyl acetate saturated alcohol solution for 8 min, observed under transmission electron microscope, and images were collected for analysis.

### 2.12. Statistical Analysis

When it comes to data analysis, we must remember that all data are nothing more than a representation of the underlying reality that we are attempting to understand. That being said, in this study, all of the data that we collected were expressed as means ± SEM, which is a standard way of presenting statistical data in the scientific community. To analyze the data, we utilized the powerful Prism 9.0 software, which is a popular choice for many researchers due to its versatility and ease of use. When comparing two groups, we utilized a two-tailed unpaired Student’s *t*-test, which is a common statistical method used to determine whether there is a significant difference between two means. The level of significance was denoted by asterisks: * *p* < 0.05, ** *p* < 0.01, *** *p* < 0.001, and **** *p* < 0.0001, which indicates the level of statistical significance of the results. So, as you can see, the statistical analysis was quite robust, and we were able to draw meaningful conclusions from the data that we collected.

## 3. Results

### 3.1. n-3 PUFA Reduces Obesity and Serum Hormone Levels in PCOS Mice

**Figure 1:**
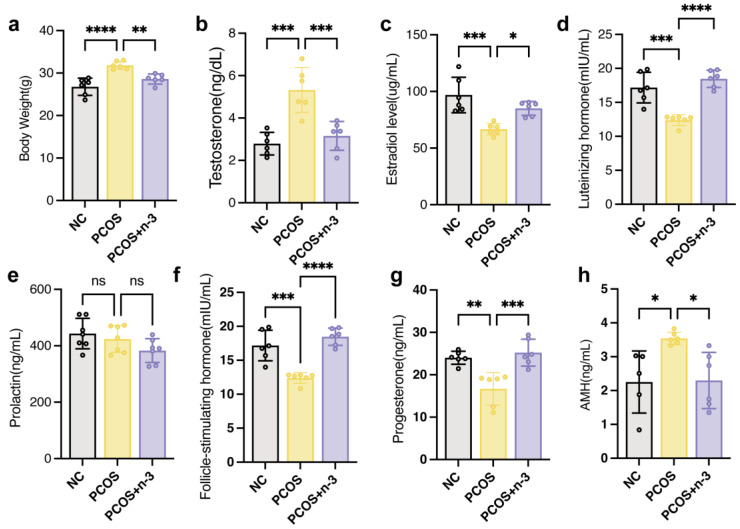
Serum hormone disorders in PCOS mice were alleviated by *n*-3 PUFA addition. (**a**) The body weight of control, PCOS, PCOS + *n*-3 PUFA group mice. (**b**–**h**) The serum T, LH, FSH, Prog and AMH content in the control group, PCOS and PCOS + *n*-3 PUFA were statistically different. The difference in the serum content of PRL was not statistically significant among the three groups. Data are means ± SD (n = 6 per group). Polycystic Ovary Syndrome (PCOS), (T, testosterone; E2, Estradiol; LH, luteinizing hormone; Prl, Prolactin; FSH, follicle stimulating hormone; Prog, Progesterone; AMH, anti-Müllerian hormone) * *p* < 0.05, ** *p* < 0.01, *** *p* < 0.001, **** *p* < 0.0001. ns represents no significant difference.

To determine the effect of *n*-3 PUFA on PCOS mice, mice successfully constructed in a 6-week-old PCOS model were gavaged with 0.2 mL each for 60 days (DHA 180 mg/mL; EPA 120 mg/mL)of *n*-3 PUFA for 2 months and changes in serum hormone levels were examined. As shown in Figure 1a, we examined the body weight of PCOS mice, and the results showed that the treatment of DHEA-induced PCOS in mice significantly contributed to their body weight. In addition, after treatment of PCOS mice with *n*-3 PUFA, we compared the serum estradiol (Figure 1c), testosterone (Figure 1b), luteinizing hormone (Figure 1d), prolactin (Figure 1e), FSH (Figure 1f), progesterone (Figure 1g), and AMH (Figure 1h) levels in the PCOS group with those in the PCOS + *n*-3 PUFA group. The results showed that the levels of hormones associated with ovarian function in the *n*-3 PUFA added mice. The results showed that *n*-3 PUFA treatment suppressed the expression of hyperandrogenism in the peripheral blood of mice, improved ovarian function, and promoted the peripheral blood levels of estradiol as well as progesterone. Similarly, anti-Müllerian hormones, which are responsive to resting follicle reserve in the mammalian ovary in adulthood, are usually significantly upregulated in PCOS patients. As shown in Figure 1h, AMH in PCOS mice was significantly reduced following the addition of *n*-3 PUFA treatment.

### 3.2. n-3 PUFA Improves Estrous Cycle and Ovarian Index in PCOS Mice

**Figure 2:**
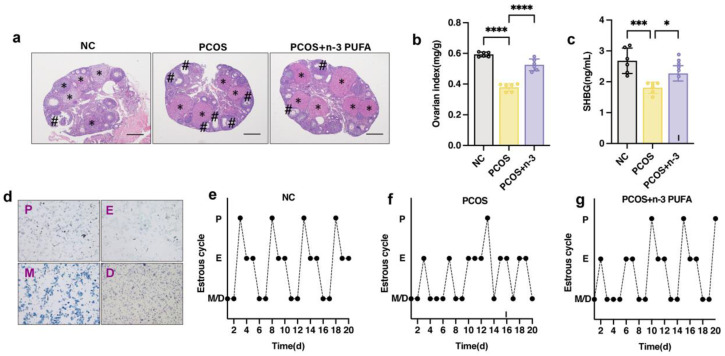
Effect of *n*-3 PUFA on ovarian tissue morphology and estrous cycle in PCOS mice. (**a**) The impact of *n*-3 PUFA on the ovary of PCOS mice was detected by the morphology of ovarian tissue detected by HE staining. (**b**) Ovarian index of mice in control, PCOS and PCOS + *n*-3 treatment groups (Ovary weight mg/Body weight g). (**c**) Serum SHBG level in control, PCOS and PCOS + *n*-3 treatment groups. (**d**) In order to accurately judge the estrus stage of mice, the vaginal epithelial cells of mice were observed by toluidine blue staining experiment, P for pre-estrus E for estrus M for post-estrus D for interestrus. (**e**–**g**) Oestrus cycle statistics of mice in control, PCOS and PCOS + *n*-3 treatment groups. Data are means ± SD (n = 6). * represents corpus luteum; # represents antral follicle. * *p* < 0.05, *** *p* < 0.001, **** *p* < 0.0001.

**Figure 3:**
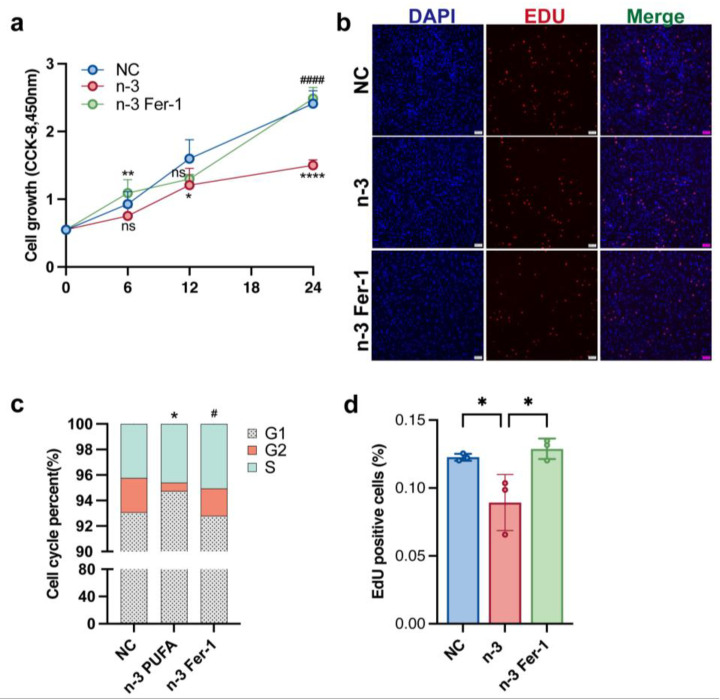
After the medium of KGN cell were added *n*-3 PUFA the CCK-8 assay, EdU assay were used to determine cell proliferation. The addition of *n*-3 PUFA significantly inhibited the proliferation of KGN cells, on the contrary, the addition of ferroptosis inhibitor Fer-1 significantly restored the proliferation ability of KGN cells. The result of CCK-8 (**a**) is consistent with the result of EDU experiment (**b**), and (**d**) is the statistical result of EDU experiment. After KGN cells were treated with *n*-3 PUFA, the results of flow cytometry showed that the number of cells in the G2 phase was significantly reduced, suggesting that *n*-3 PUFA inhibited cell proliferation (**c**). Data are means ± SD (n = 3). * *p* < 0.05, ** *p* < 0.01, **** *p* < 0.0001, # *p* < 0.05, #### *p* < 0.0001. ns represents no significant difference.

Polycystic OVARY SYNDROME (PCOS) is a complex condition that manifests as enlarged ovaries and polycystic lesions in patients. In order to investigate the effects of dietary *n*-3 polyunsaturated fatty acid (PUFA) addition in PCOS mice, we measured their ovarian index and compared it to a control group as depicted in Figure 2a. Additionally, we sought to confirm the positive impact of *n*-3 PUFA on PCOS mice by evaluating their oestrus cycle, ovarian index, and histomorphology, as illustrated in Figure 2b. Moreover, we explored the potential of sex hormone binding globulin (SHBG), a sex hormone carrier and a marker for PCOS, to regulate PCOS symptoms. As demonstrated in Figure 2c, *n*-3 PUFA significantly increased SHBG levels in the blood of PCOS mice, indicating the therapeutic potential of *n*-3 PUFA in PCOS treatment.

To further investigate the effects of *n*-3 PUFA on PCOS, we monitored the mice for a 14-day kinetic cycle using toluidine blue staining of vaginal epithelial cells. The examination revealed that PCOS mice lacked a regular estrous cycle and had an extended interestrous period compared to the control group. However, the *n*-3 PUFA-treated group showed significant restoration of the disturbed estrous cycle of the mice, as evidenced by the vaginal epithelial examination shown in Figure 2d–g.

In the PCOS group, mice exhibited polycystic ovaries with a reduced number of corpora luteum and an increased number of luminal follicles, with no dominant follicles observed. Conversely, the *n*-3 PUFA-treated group showed an increase in the number of corpora luteum, and improved ovarian function was observed histomorphologically in the mice.

Overall, these results demonstrate the potential of *n*-3 PUFA as a therapeutic strategy for PCOS management. The data also highlight the importance of carefully considering the impact of diet and lifestyle factors on the development and progression of PCOS.

### 3.3. n-3 PUFA Inhibits the Proliferation of Ovarian Granulosa Cells In Vitro

Since the pathogenesis of polycystic ovaries is associated with abnormally proliferating ovarian granulosa cells, we investigated the role of *n*-3 PUFA in regulating the proliferation of ovarian granulosa cells. *n*-3 PUFA is a conventional in vitro experimental model of ovarian granulosa cells. Ferrostatin-1, a synthetic antioxidant that inhibits cell death by protecting against membrane lipid damage, is an iron death inhibitor commonly applied in in vitro experimental studies. To verify the effect of *n*-3 PUFA on KGN cells, we added a group of experimental groups treated with *n*-3 PUFA combined with fer-1 to see whether the addition of Fer-1 could weaken the inhibitory effect of *n*-3 PUFA on the occurrence of cellular iron death. The results shown in Figure 3b,d demonstrate that the treatment of KGN cells with *n*-3 PUFA significantly inhibited the proliferative capacity of the cells, and the EDU results were consistent with those with CCK-8 (Figure 3a). The above results imply to us that *n*-3 PUFA treatment of KGN cells can significantly inhibit cell proliferation. The results of the flow cytometric cell cycle assay also demonstrated the inhibitory effect of *n*-3 PUFA on the proliferative capacity of KGN cells (Figure 3d).

### 3.4. n-3 PUFA Promotes Ferroptosis Formation in Ovarian Granulosa Cells

We performed tunnel staining of mouse ovaries from PCOS, PCOS + *n*-3 PUFA, and control groups in an attempt to elucidate the mechanism by which *n*-3 PUFA inhibits ovarian granulosa cell proliferation.

**Figure 4:**
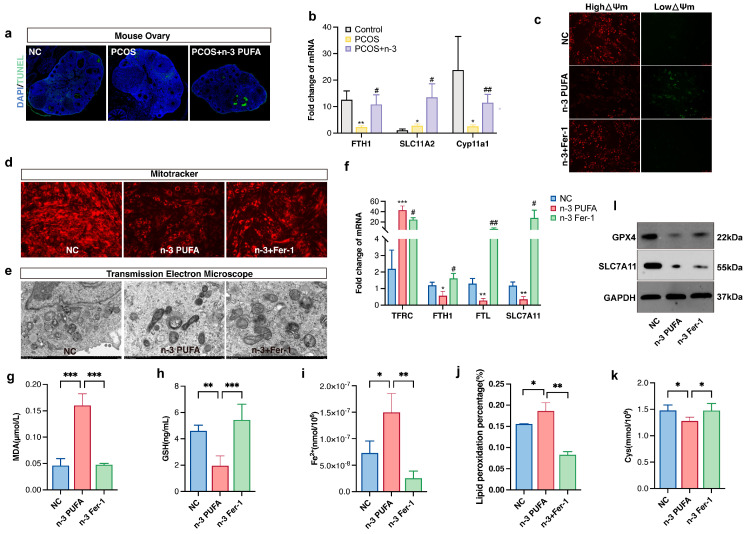
(**a**) TUNEL immunofluorescence staining of mouse ovarian tissue (**b**) Ferroptosis marker gene expression in control, PCOS and PCOS + *n*-3 PUFA group mice ovary. (**c**) JC-1 assay to detect mitochondrial membrane potential of KGN cell determining whether mitochondrial function is impaired, and (**d**) mitotracker stain assay to detect mitochondrial activity in control, *n*-3 and *n*-3 + Fer-1 group. (**e**) Transmission electron microscopy scan to observe the extent of cellular iron death, compared with the control group, the mitochondria in the *n*-3 treatment group were pyknotic, and the mitochondrial cristae were not clear, and the mitochondria were ruptured. However, after adding the ferroptosis inhibitor Fer-1, the damaged mitochondrial morphology was partially restored. In addition, in order to further confirm the effect of *n*-3 PUFA on KGN cells, we used qRT-PCR to detect the expression of ferroptosis marker genes (**f**), the content of MDA (**g**), the content of reduced glutathione (**h**), and the content of Fe^2+^ (**i**) and LPO (**j**) cysteine (**k**) in control, *n*-3 and *n*-3 + Fer-1 treatment groups. (**l**) The expression levels of GPX4 and SLC7A11 proteins were detected in control, *n*-3 and *n*-3 + Fer-1 treatment groups. Data are means ± SD (n = 3). * *p* < 0.05, ** *p* < 0.01, *** *p* < 0.001, # *p* < 0.05, ## *p* < 0.01.

As shown in Figure 4a, the ovary of mice in the *n*-3 PUFA group displayed a conspicuous increase in apoptotic granulosa cell signaling, which led us down a rabbit hole of further investigation. We determined that ferroptosis was the culprit, a phenomenon that we attributed to an increase in MDA due to lipid peroxidation and a decrease in reduced glutathione, along with the oxidation of iron ions to ferrous ions, which disrupted mitochondrial function and caused non-programmed cell death. In order to gain a deeper understanding of these findings, we scrutinized the concentrations of reduced glutathione, MDA (Figure 4g), Fe^2+^ (Figure 4i), and GSH (Figure 4h), as well as the expression of ferroptosis marker genes (Figure 4b) in ovarian tissues from the aforementioned treatment groups.

As we delved deeper into our perplexing results, we observed that the expression of TFRC and ACSL4 was significantly upregulated in the *n*-3 PUFA-treated group compared to the PCOS group, while the expression trends of SLC7A11, FTH, and FTL were flipped on their heads (Figure 4f). In an effort to verify the effects of *n*-3 PUFA on PCOS GCs, we conducted in vitro level experiments with mitotracker (Figure 4d) and JC-1 staining (Figure 4c) to observe changes in mitochondrial activity, as well as membrane potential. The findings were astounding: *n*-3 PUFA had a profound inhibitory effect on the mitochondrial activity of GCs and was also found to promote a shift from high to low mitochondrial membrane potential.

To add to the already overwhelming complexity of our results, the experimental findings of bodipy581/591 (Figure 4j), a lipophilic fluorescent probe for detecting lipid peroxidation in cell membranes, were consistent with the trends observed in the JC-1 experiments. We then conducted projection electron microscopy scans to further confirm that *n*-3 PUFA induced ferroptosis in KGN cells. The results were unequivocal: *n*-3 PUFA caused mitochondrial sequestration and an increase in the density of bilayer structures in KGN cells, consistent with the ultrastructure of cells undergoing ferroptosis. We also discovered that the regulatory effect of *n*-3 could be rescued by fer-1, which is a ferroptosis inhibitor.

Lastly, we determined the amount of Fe^2+^ and the expression of transferrin in *n*-3 PUFA treated cells, which was of vital importance to our investigation. Our findings were nothing short of confounding: *n*-3 PUFA increased the amount of intracellular ferrous ions while simultaneously decreasing the amount of intracellular transferrin. The implications of these results are staggering and may have far-reaching consequences for the field of ovarian granulosa cell proliferation research. Cysteine plays an important role in the xc-system, and we found that the content of cysteine was significantly reduced after adding *n*-3 PUFA to KGN medium. In addition, our study showed that the expression of SLC7A11, a key regulator of the xc-system, also decreased after *n*-3 PUFA treatment, and the protein expression of GPX4 also showed the same trend.

### 3.5. n-3 PUFA Promotes Ferroptosis through Hippo Pathway

**Figure 5:**
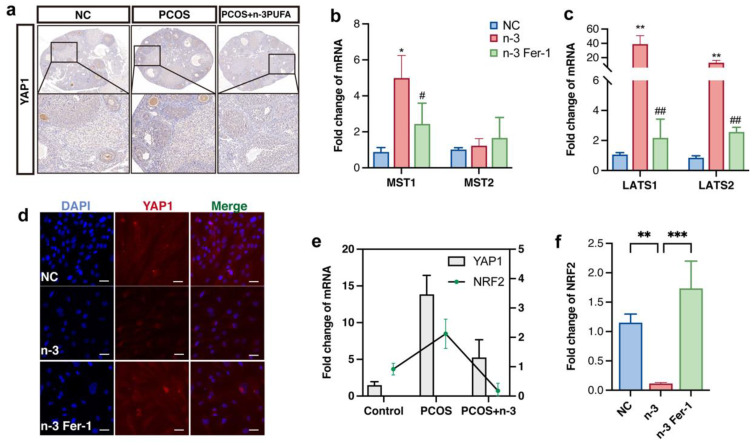
(**a**) Immunohistochemical staining of YAP1 in control, PCOS and PCOS + *n*-3 PUFA group mice ovary. *n*-3 PUFA addition group significantly decreased the expression of YAP1, implying an increase in p-YAP1. (**b**,**c**) Gene expression upstream of Hippo pathway which located upstream genes involved in the regulation of YAP1 phosphorylation. (**d**) Immunofluorescence staining of YAP1, after activation of the Hippo pathway, Yap1 is phosphorylated in the cytoplasm, and its total amount and nuclear distribution are reduced. (**e**) The expression trend of YAP1 and NRF2 was the same in Control, PCOS, PCOS + *n*-3 PUFA ovarian tissues (**f**) Further detection of the expression trends of NRF2 in vitro experiments showed that NRF2 were significantly down-regulated in the *n*-3 PUFA treatment group compared with the control group. Data are means ± SD (n = 3). * *p* < 0.05, ** *p* < 0.01, *** *p* < 0.001, # *p* < 0.05, ## *p* < 0.01.

Utilizing immunohistochemical staining as a means to detect YAP1 protein expression within each group of mice models, our findings indicate a significant reduction in total YAP1 protein content following PCOS + *n*-3 PUFA treatment as compared with the PCOS group, as illustrated in Figure 5a. It is essential to note that the Hippo pathway plays an integral role in a myriad of biological processes, including cell proliferation, organ size, and mechanical stress [21]. When the resting Hippo pathway is activated, YAP1 is intercepted and phosphorylated outside the nucleus for degradation. Therefore, our study sought to examine the nuclear-cytoplasmic distribution of YAP1 in KGN cells treated with *n*-3 PUFA compared with the control group, which revealed a marked reduction in the number of YAP1 nuclei in the *n*-3 PUFA group as opposed to the PCOS group, as displayed in Figure 5b.

To validate our hypothesis that *n*-3 PUFA activates ferroptosis marker genes by inhibiting the Hippo pathway’s role in promoting yap1 entry into the nucleus, we evaluated the expression of upstream kinases of the Hippo signaling pathway in a mouse model. Our findings demonstrated that the expression of LAST1/2 MST1/2 was significantly downregulated in the *n*-3 PUFA-treated group, as illustrated in Figure 5b,c. Furthermore, we conducted cellular level validation experiments in KGN cell lines, which yielded similar results in that the nuclei distribution of YAP was significantly reduced in *n*-3 treated cells.

Prior research has established the critical role of YAP1 in ferroptosis, specifically inhibiting the onset of ferroptosis through crosstalk with NRF2. Thus, *n*-3 PUFA may promote ferroptosis in GCs that proliferate abnormally in PCOS by encouraging the out-of-cell nuclear translocation of YAP1. Interestingly, consistent with previous research, the expression trend of nrf2 mirrored that of YAP1, indicating crosstalk between YAP1 and NRF2, as mentioned in earlier studies. Additionally, NRF2 expression was significantly down-regulated following *n*-3 PUFA treatment and up-regulated following the inclusion of ferroptosis inhibitors.

## 4. Discussion

Polycystic ovary syndrome affects 4–12% of women of childbearing age who fail to conceive normally [4]. There are many factors that contribute to the development of polycystic ovaries, including centripetal obesity, genetic factors, and environmental factors [4]. There are no specific medications available to treat polycystic ovary syndrome, and the existing treatment options such as clomiphene and letrozole, as well as laparoscopic ovarian drilling, have some drawbacks [6]. For example, the incidence of fertility defects is significantly higher in patients treated with letrozole, while surgical relief of PCOS is associated with complications of adnexal adhesions, particularly in obese PCOS patients [3].

Intriguingly, an increasing number of studies have shown that the addition of *n*-3 PUFA to the diet has a positive effect on the recovery of patients with PCOS. PCOS is characterized by hyperandrogenemia and associated insulin resistance. Approximately 75% of patients with PCOS are insulin resistant. There are numerous reports that *n*-3 PUFA can alleviate insulin resistance in PCOS patients. In addition, *n*-3 PUFA can improve dyslipidemia, chronic inflammatory environment, increase sex hormone binding globulin and reduce free androgen index in PCOS patients. However, few studies have shown the molecular mechanisms underlying the positive effects of *n*-3 PUFA on PCOS patients.

Recent studies on the effects of *n*-3 PUFA on ovarian granulosa cells have shown that *n*-3 PUFA can inhibit the proliferation of ovarian granulosa cells and alleviate follicular arrest due to abnormally proliferating cells [22]. However, most of these studies have been conducted at the clinical level or only at the cellular level, and no specific work has been conducted to investigate the molecular mechanisms in vivo and in cells.

The enigmatic nature of PCOS is exemplified by the fact that follicle development is stuck at the small or medium-sized sinusoidal follicle stage, and the molecular mechanisms of developmental arrest are not clear [26]. Ovarian granulosa cells, which are important regulators of follicular development, perform different physiological functions in the development of the follicle, atresia, and ovulation [27]. The Hippo signaling pathway is thought to be a signaling pathway associated with the onset of mechanical stress in the outside world of the cell. During the lutealisation phase of the follicle, the Hippo signaling pathway was activated, which inhibits the regulatory role of YAP1 in the nucleus, thereby promoting luteinisation of ovarian granulosa cells, ultimately in response to LH regulation. Thus, normal activation of the Hippo signaling pathway is necessary during the terminal differentiation phase of the follicle.

The complexity of PCOS is further highlighted by the increased ovarian size in PCOS compared to normal patients, which appears to be due to the loss of the ability of cortical cells and granulosa cells in the ovary to regulate normal apoptosis and proliferation associated with abnormal expression of the Hippo signaling pathway. Ferroptosis of cells is a non-programmed cell death due to phospholipid peroxidation of the cell membrane. Glutathione, reactive oxygen species, and unsaturated fatty acids can all regulate cellular ferroptosis. Yap1 has been reported to crosstalk with NRF2 in cancer development, regulating the susceptibility of cancer cells to ferroptosis [24]. In addition, NRF2 is a key regulator of resistance to oxidative stress. Under normal conditions, Keap1 protein interacts with and retains Nrf2 in the cytoplasm, and Keap1 keeps Nrf2 at low levels by mediating its ubiquitinated degradation [28,29].

Mechanistically, the study found that *n*-3 PUFA inhibited the nuclear entry of YAP1 by activating the Hippo signal pathway in the PCOS model, while inhibiting the crosstalk between YAP1 and NRF2, ultimately promoting ferroptosis in ovarian granulosa cells. These findings suggest that *n*-3 PUFA could be a promising therapeutic option for PCOS patients by targeting the Hippo-YAP1-NRF2-ferroptosis axis.

In conclusion, PCOS is a complex endocrine disorder affecting a significant percentage of women of childbearing age [30]. While there are limited treatment options available, recent studies have suggested that the addition of *n*-3 PUFA to the diet could have a positive effect on PCOS patients. The present study provides new insights into the molecular mechanisms underlying the beneficial effects of *n*-3 PUFA on PCOS, specifically through the activation of the Hippo pathway, inhibition of YAP1 nuclear entry, and promotion of ferroptosis in ovarian granulosa cells. These findings offer a promising new avenue for the development of targeted therapies for PCOS.

There are many reports on the positive effects of *n*-3 PUFA in the treatment of PCOS. Ling et al. [31]. used multivariate linear regression models to analyze the relationship between dietary and serum omega-3 fatty acids and homeostasis model assessment of insulin resistance (HOMA-IR) levels and body composition parameters in patients with PCOS. The results indicated that the addition of *n*-3 PUFA to the diet had a beneficial effect on metabolic parameters and body composition in PCOS patients. Komal et al. [32]. showed that omage-3 PUFA had a positive effect on the improvement of lipid metabolism and hormonal parameters in the blood of PCOS rats, as well as on insulin sensitivity and inflammation. Most patients with polycystic ovaries are characterized by abdominal fat accumulation or centripetal obesity, which eventually leads to decreased insulin sensitivity and androgenemia. This poor physiological environment affects the metabolic and reproductive functions of PCOS patients, and impaired angiogenesis and endothelial dysfunction are also caused. A study conducted by Mehri et al. [33]. showed that the addition of *n*-3 PUFA to the diet of patients with PCOS had a positive effect on improving their mental health and serum levels of androgens. The above results are the same as those obtained in our experiments, where the addition of *n*-3 PUFA to the diet of mice led to a slight reduction in body weight and alleviated hyperandrogenemia. The lipid metabolism in the blood and the molecular regulatory mechanisms need to be further elucidated.

In addition, our study sheds light on the potential molecular mechanisms underlying the positive effects of *n*-3 PUFA in PCOS treatment. Our findings suggest that *n*-3 PUFA can activate the Hippo signaling pathway, which in turn inhibits the nuclear entry of YAP1 and promotes ferroptosis in ovarian granulosa cells. This provides a potential explanation for the observed inhibitory effect of *n*-3 PUFA on the proliferation of ovarian granulosa cells and the alleviation of follicular arrest due to abnormally proliferating cells.

Overall, our study highlights the potential of *n*-3 PUFA as a novel therapeutic approach for the treatment of PCOS. Our findings suggest that the beneficial effects of *n*-3 PUFA may be mediated through the activation of the Hippo signaling pathway and the promotion of ferroptosis in ovarian granulosa cells. Further studies are needed to confirm our findings and to explore the clinical implications of *n*-3 PUFA supplementation in PCOS patients. Nevertheless, our study provides a new research perspective for the treatment of PCOS and may pave the way for the development of new therapeutic strategies for this common endocrine disorder.

## 5. Conclusions

Our current study serves to elucidate the efficaciousness of incorporating *n*-3 PUFA into the dietary regimen as a potential palliative intervention for polycystic ovary syndrome (PCOS). Specifically, our findings demonstrate that *n*-3 PUFA operates to impede the proliferation of ovarian granulosa cells through the suppression of nuclear translocation of YAP1 via the activation of the Hippo signaling pathway. This suppressive mechanism is further amplified by the concurrent inhibition of NRF2, a protein that governs the regulation of critical genes involved in ferroptosis, ultimately leading to the promotion of ferroptosis in ovarian granulosa cells. Our research thus furnishes groundbreaking insight into the governing mechanisms underlying the incorporation of moderate amounts of *n*-3 PUFA into the diet as a potential intervention for the clinical remission of PCOS.

## Data Availability

The datasets used or analyzed during the current study are available from the corresponding author upon reasonable request.

## Supplementary Materials

The following supporting information can be downloaded at: https://www.mdpi.com/article/10.3390/nu15081927/s1, Table S1: The primer sequences used for qRT-PCR.

## Abbreviations

PCOS	Polycystic ovary syndrome
PUFA	Polyunsaturated fatty acid
SNP	Single Nucleotide Polymorphism
NRF2	Nuclear factor erythroid2-related factor 2
ROS	Reactive oxygen species
NAFLD	Non-alcoholic fatty liver disease
CCK8	Cell Counting Kit-8
MDA	Malondialdehyde
TFRC	Transferrin receptor
GSH	Glutathione
ACSL4	Acyl-CoA synthetase member of the long chain Family 4
SLC7A11	Recombinant Solute Carrier Family 7, Member 11
FTH	Ferritin heavy polypeptide 1
FTL	Ferritin light polypeptide
MST1	Macrophage Stimulating 1
MST2	Macrophage Stimulating 2
LATS1	Large tumor suppressor kinase 1
LATS2	Large tumor suppressor kinase 2

## References

[1] Patel S. (2018). Polycystic ovary syndrome (PCOS), an inflammatory, systemic, lifestyle endocrinopathy. J. Steroid Biochem. Mol. Biol..

[2] Burnatowska E., Wikarek A., Oboza P., Ogarek N., Glinianowicz M., Kocelak P., Olszanecka-Glinianowicz M. (2023). Emotional Eating and Binge Eating Disorders and Night Eating Syndrome in Polycystic Ovary Syndrome-A Vicious Circle of Disease: A Systematic Review. Nutrients.

[3] Palioura E., Diamanti-Kandarakis E. (2015). Polycystic ovary syndrome (PCOS) and endocrine disrupting chemicals (EDCs). Rev. Endocr. Metab. Disord..

[4] Gu Y., Zhou G., Zhou F., Wu Q., Ma C., Zhang Y., Ding J., Hua K. (2022). Life Modifications and PCOS: Old Story But New Tales. Front. Endocrinol..

[5] Naderpoor N., Shorakae S., de Courten B., Misso M.L., Moran L.J., Teede H.J. (2015). Metformin and lifestyle modification in polycystic ovary syndrome: Systematic review and meta-analysis. Hum. Reprod. Update.

[6] Liao B., Qiao J., Pang Y. (2021). Central Regulation of PCOS: Abnormal Neuronal-Reproductive-Metabolic Circuits in PCOS Pathophysiology. Front. Endocrinol..

[7] Dapas M., Dunaif A. (2022). Deconstructing a Syndrome: Genomic Insights Into PCOS Causal Mechanisms and Classification. Endocr. Rev..

[8] Salek M., Clark C.C.T., Taghizadeh M., Jafarnejad S. (2019). N-3 fatty acids as preventive and therapeutic agents in attenuating PCOS complications. EXCLI J..

[9] Alesi S., Ee C., Moran L.J., Rao V., Mousa A. (2022). Nutritional Supplements and Complementary Therapies in Polycystic Ovary Syndrome. Adv. Nutr..

[10] Barrea L., Arnone A., Annunziata G., Muscogiuri G., Laudisio D., Salzano C., Pugliese G., Colao A., Savastano S. (2019). Adherence to the Mediterranean Diet, Dietary Patterns and Body Composition in Women with Polycystic Ovary Syndrome (PCOS). Nutrients.

[11] Tapiero H., Ba G.N., Couvreur P., Tew K.D. (2002). Polyunsaturated fatty acids (PUFA) and eicosanoids in human health and pathologies. Biomed. Pharmacother..

[12] Broughton K.S., Rule D.C., Ye Y., Zhang X., Driscoll M., Culver B. (2009). Dietary omega-3 fatty acids differentially influence ova release and ovarian cyclooxygenase-1 and cyclooxygenase-2 expression in rats. Nutr. Res..

[13] Li J., Cao F., Yin H.L., Huang Z.J., Lin Z.T., Mao N., Sun B., Wang G. (2020). Ferroptosis: Past, present and future. Cell Death Dis..

[14] Sun Y., Chen P., Zhai B., Zhang M., Xiang Y., Fang J., Xu S., Gao Y., Chen X., Sui X. (2020). The emerging role of ferroptosis in inflammation. Biomed. Pharmacother..

[15] Chen X., Kang R., Kroemer G., Tang D. (2021). Ferroptosis in infection, inflammation, and immunity. J. Exp. Med..

[16] Su Y., Zhao B., Zhou L., Zhang Z., Shen Y., Lv H., AlQudsy L.H.H., Shang P. (2020). Ferroptosis, a novel pharmacological mechanism of anti-cancer drugs. Cancer Lett..

[17] Dierge E., Debock E., Guilbaud C., Corbet C., Mignolet E., Mignard L., Bastien E., Dessy C., Larondelle Y., Feron O. (2021). Peroxidation of *n*-3 and n-6 polyunsaturated fatty acids in the acidic tumor environment leads to ferroptosis-mediated anticancer effects. Cell Metab..

[18] Chen J., Zaal E.A., Berkers C.R., Ruijtenbeek R., Garssen J., Redegeld F.A. (2021). Omega-3 Fatty Acids DHA and EPA Reduce Bortezomib Resistance in Multiple Myeloma Cells by Promoting Glutathione Degradation. Cells.

[19] Brand A., Schonfeld E., Isharel I., Yavin E. (2008). Docosahexaenoic acid-dependent iron accumulation in oligodendroglia cells protects from hydrogen peroxide-induced damage. J. Neurochem..

[20] Jump D.B., Lytle K.A., Depner C.M., Tripathy S. (2018). Omega-3 polyunsaturated fatty acids as a treatment strategy for nonalcoholic fatty liver disease. Pharmacol. Ther..

[21] Maas K., Mirabal S., Penzias A., Sweetnam P.M., Eggan K.C., Sakkas D. (2018). Hippo signaling in the ovary and polycystic ovarian syndrome. J. Assist. Reprod. Genet..

[22] Clark K.L., George J.W., Przygrodzka E., Plewes M.R., Hua G., Wang C., Davis J.S. (2022). Hippo Signaling in the Ovary: Emerging Roles in Development, Fertility, and Disease. Endocr. Rev..

[23] Lidaka L., Bekere L., Lazdane G., Lazovska M., Dzivite-Krisane I., Gailite L. (2022). Role of Single Nucleotide Variants in the YAP1 Gene in Adolescents with Polycystic Ovary Syndrome. Biomedicines.

[24] Yang Y., Li X., Wang J., Tan J., Fitzmaurice B., Nishina P.M., Sun K., Tian W., Liu W., Liu X. (2021). A missense mutation in Pitx2 leads to early-onset glaucoma via NRF2-YAP1 axis. Cell Death Dis..

[25] Zhang D., Yi S., Cai B., Wang Z., Chen M., Zheng Z., Zhou C. (2021). Involvement of ferroptosis in the granulosa cells proliferation of PCOS through the circRHBG/miR-515/SLC7A11 axis. Ann. Transl. Med..

[26] Mork L., Maatouk D.M., McMahon J.A., Guo J.J., Zhang P., McMahon A.P., Capel B. (2012). Temporal differences in granulosa cell specification in the ovary reflect distinct follicle fates in mice. Biol. Reprod..

[27] McFee R.M., Romereim S.M., Snider A.P., Summers A.F., Pohlmeier W.E., Kurz S.G., Cushman R.A., Davis J.S., Wood J.R., Cupp A.S. (2021). A high-androgen microenvironment inhibits granulosa cell proliferation and alters cell identity. Mol. Cell. Endocrinol..

[28] Tao G., Kahr P.C., Morikawa Y., Zhang M., Rahmani M., Heallen T.R., Li L., Sun Z., Olson E.N., Amendt B.A. (2016). Pitx2 promotes heart repair by activating the antioxidant response after cardiac injury. Nature.

[29] Dai H., Wei Y., Liu Y., Liu J., Yu R., Zhang J., Pang J., Shao Y., Li Q., Yang Z. (2021). Pathway-Based Analysis Revealed the Role of Keap1-Nrf2 Pathway and PI3K-Akt Pathway in Chinese Esophageal Squamous Cell Carcinoma Patients with Definitive Chemoradiotherapy. Front. Genet..

[30] Zhang Y., Ho K., Keaton J.M., Hartzel D.N., Day F., Justice A.E., Josyula N.S., Pendergrass S.A., Actkins K., Davis L.K. (2020). A genome-wide association study of polycystic ovary syndrome identified from electronic health records. Am. J. Obstet. Gynecol..

[31] Lu L., Li X., Lv L., Xu Y., Wu B., Huang C. (2022). Associations between omega-3 fatty acids and insulin resistance and body composition in women with polycystic ovary syndrome. Front. Nutr..

[32] Komal F., Khan M.K., Imran M., Ahmad M.H., Anwar H., Ashfaq U.A., Ahmad N., Masroor A., Ahmad R.S., Nadeem M. (2020). Impact of different omega-3 fatty acid sources on lipid, hormonal, blood glucose, weight gain and histopathological damages profile in PCOS rat model. J. Transl. Med..

[33] Jamilian M., Samimi M., Mirhosseini N., Afshar Ebrahimi F., Aghadavod E., Talaee R., Jafarnejad S., Hashemi Dizaji S., Asemi Z. (2018). The influences of vitamin D and omega-3 co-supplementation on clinical, metabolic and genetic parameters in women with polycystic ovary syndrome. J. Affect. Disord..

