# Supplementary material for: n-3 PUFA Promotes Ferroptosis in PCOS GCs by Inhibiting YAP1 through Activation of the Hippo Pathway
Source: Nutrients. 2023 Apr 16;15(8):1927. doi: 10.3390/nu15081927 (PMC10145554; doi:10.3390/nu15081927)
Supplement: Supplementary file 1 [file nutrients-15-01927-s001.zip › Supplementary Table S1.pdf]

Gene	Primer sequence (5`→3`)
TFRC	F:ACCATTGTCATATACCCGGTTCA<br>R:CAATAGCCCAAGTAGCCAATCAT
ACSL4	F:CATCCCTGGAGCAGATACTCT<br>R:TCACTTAGGATTTCCCTGGTCC
SLC7A11	F:TCTCCAAAGGAGGTTACCTGC<br>R:AGACTCCCCTCAGTAAAGTGAC
FTH	F:ACTTTGACCGCGATGATGTG<br>R:GCTCTCCCAGTCATCACAGT
FTL	F:ATGGGGTGCGGACTTAGAAAG<br>R:CTTGCGGTCTCTTCAGGGTAG
MST1	F:CAAGGTCTGGACGACAACTATTG<br>R:CAGTGGTGGTATTGGCTGTG
MST2	F:CGATGTTGGAATCCGACTTGG<br>R:GTCTTTGTACTTGTGGTGAGGTT
LATS1	F: TTACCAAGATCCTCGACGAGAG<br>R:CACATTCCCTGGTTTCATGCT
LATS2	F: ACTTTTCCTGCCACGACTTATTC<br>R:GATGGCTGTTTTAACCCCTCA
NRF2	F:TTCCCGGTCACATCGAGAG<br>R:TCCTGTTGCATACCGTCTAAATC
β-actin	F:CATGTACGTTGCTATCCAGGC<br>R: CTCCTTAATGTCACGCACGAT

Supplementary Table S1. The primer sequences used for qRT-PCR. F: forward, R: reverse. U6 and *β-actin* were used as endogenous control genes for miRNA and mRNA, respectively.
