# Supplementary figures and images for: n-3 PUFA Promotes Ferroptosis in PCOS GCs by Inhibiting YAP1 through Activation of the Hippo Pathway
Source: Nutrients. 2023 Apr 16;15(8):1927. doi: 10.3390/nu15081927 (PMC10145554; doi:10.3390/nu15081927)

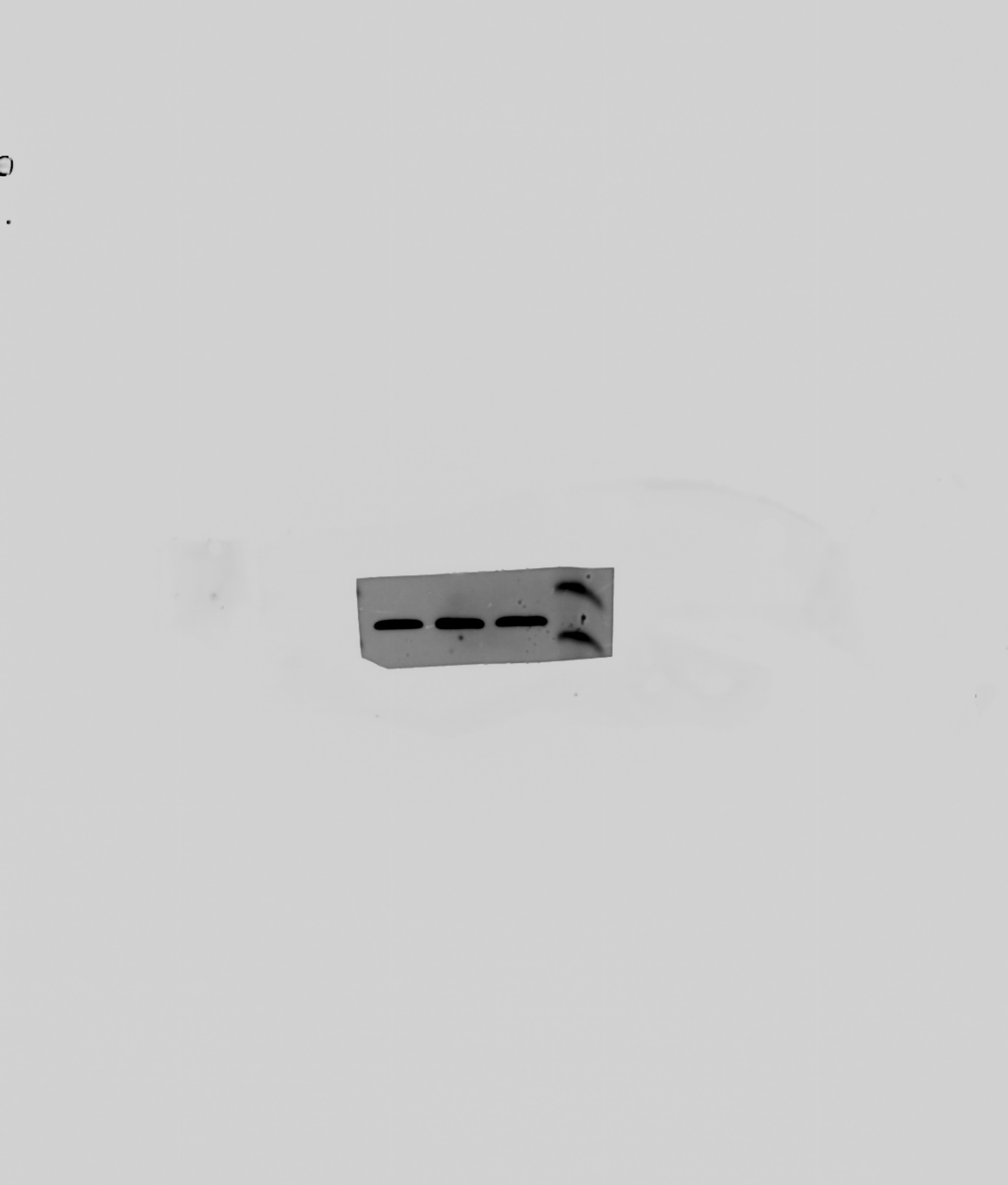

Supplement: Supplementary file 1 [file nutrients-15-01927-s001.zip › gapdh.tif]

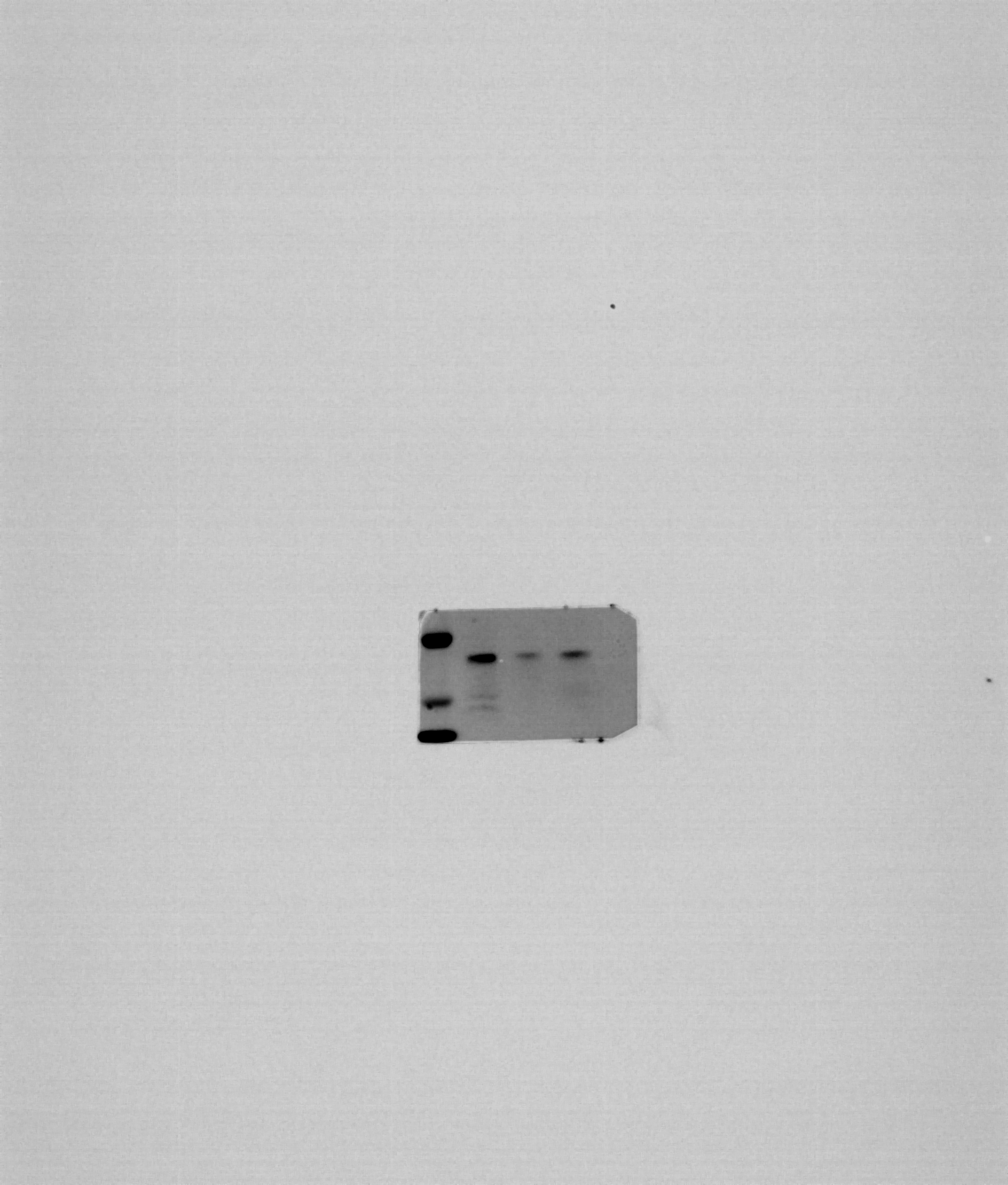

Supplement: Supplementary file 1 [file nutrients-15-01927-s001.zip › gpx4.tif]

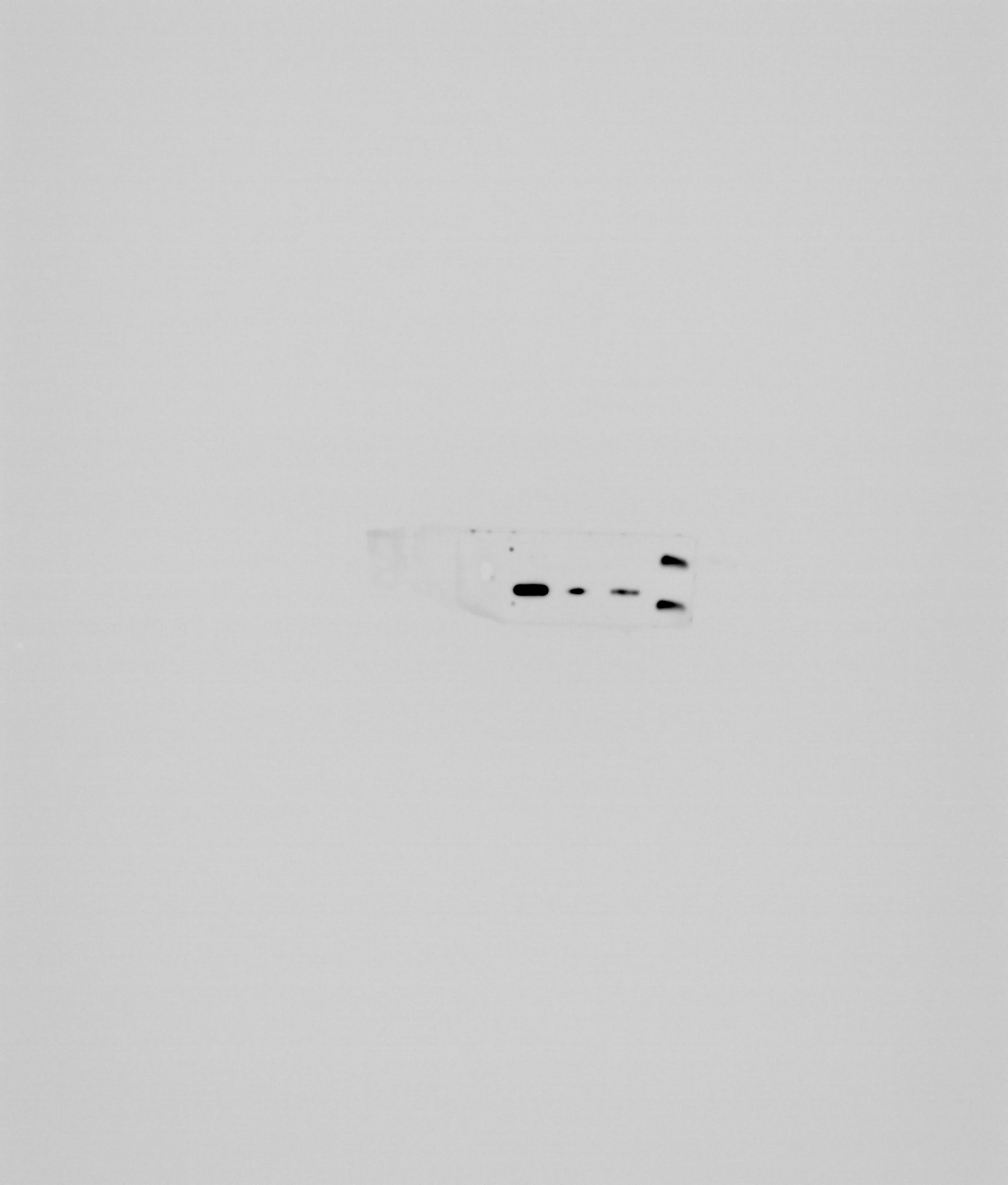

Supplement: Supplementary file 1 [file nutrients-15-01927-s001.zip › slc7a11.tif]
